# Supplementary material for: Differential Urinary Microbiome and Its Metabolic Footprint in Bladder Cancer Patients Following BCG Treatment
Source: Int J Mol Sci. 2024 Oct 17;25(20):11157. doi: 10.3390/ijms252011157 (PMC11508893; doi:10.3390/ijms252011157)
Supplement: Supplementary file 1 [file ijms-25-11157-s001.zip › #Supplementary data.pdf]

SUPPLEMENTARY DATA

Detailed methodology

Library preparation

An initial amount of 2 ng of genomic DNA was PCR amplified using Herculase II fusion DNA polymerase (Agilent Technologies, Santa Clara, CA), 5x reaction buffer, a 1mM dNTP mix, and 500nM of each of the universal F/R PCR primers. PCR was carried out with initial heat activation for 3 min at 95°C, followed by 25 cycles of 30 sec at 95°C for denaturation, 30 sec at 55°C for annealing, and 30 sec at 72°C for extension, and a final extension for 5 min at 72°C. The PCR products were then purified using AMPure beads (Agencourt Bioscience, Beverly, MA). Next, 10 μl of the first-round PCR products were further amplified for final library construction with NexteraXT Indexed Primers, under the same PCR conditions, except for using only 10 cycles. The final PCR products were purified again with AMPure beads, followed by library quantification using qPCR, according to the KAPA Library Quantification Kit for Illumina Sequencing platforms protocol, and quality assessment using TapeStation D1000 ScreenTape (Agilent Technologies, Waldbronn, Germany).

Supplementary Tables

Table S1. List of metabolites enriched in benign control and BCa groups from the external dataset by Niziol et al. [29].

Enriched in the control group (N=100)	Enriched in the cancer group (N=100)
Isovalerylcarnitine	2-Mercaptobenzothiazole
Picolinuric acid	Gamma-Glutamylcysteine
1,3-Dimethyluric acid	N-Alpha-acetyllysine
Salicyluric acid	4-Ethylphenol
2-Furoylglycine	L-Thyronine
Leu-Asp	Oleamide
Methylhippuric acid	L-Tryptophan
Indoleacetic acid	
Xanthurenic acid	
4-Methoxyphenylacetic acid	
3-Methylglutarylcarnitine	
Benzaldehyde	
3-Hydroxy-4-methoxycinnamic aicd	
Azelaic acid	
Cis,cis-Muconic acid	
2-Hydroxycaproic acid	
2-Furoic acid	
1,3,7-Trimethyluric acid	
Sebacic acid	
Hippuric acid	
Tiglylglycine	
N-Acetylserotonin	
Choline	
Phenylacetylglycine	
2,5-Furandicarboxylic acid	
3-Hydroxymandelic acid	
Acetylcysteine	
Methylmalonic acid	
Homovanillic acid	
Tetrahydroharman-3-carboxylic acid	
Phenylpropanolamine	
Dopamine	
Isostearic acid	
p-Octopamine	

3-Hydroxymethylglutaric acid
PALMITIC ACID
N-Acetyl-L-tyrosine
2-Ketohexanoic acid
Anserine
Pantothenic acid
L-Acetylcarnitine
2-Aminonicotinic acid
Phenylglyoxylic acid
N-Acetyl-L-cysteine

Table S2. Comparison of responders and non-responders for the pre-BCG samples from patients with BCa (N=29)

	Responders (n=23)	Non-responders (n=6)	*p* value
Age (yrs), median (IQR)	71 (58, 79)	80.5 (73.75, 85)	0.0684[a]
Antibiotics usage (1 month)	3 (13.0%)	0 (0.0%)	>0.9999[c]
Smoking	14 (60.0%)	3 (50.0%)	0.6693[c]
Gender, n (%)			0.3768[c]
Male	22 (95.7%)	5 (83.3%)	
Female	1(4.3%)	1 (16.7%)	
Grade, n (%)			0.5153[c]
Low	2 (8.7%)	1 (16.7%)	
High	21 (91.3%)	5 (83.3%)	
Recurrence, n (%)			**<0.0001**[c]
Recurred	0	6 (100%)	
Not recurred	23 (100%)	0	
Progression, n (%)			>0.9999[c]
Progressed	0	0	
Not progressed	23 (100%)	6 (100%)	

* Statistical methods

[a] Unpaired parametric t-test

[b] Mann-Whitney U test

[c] Fisher's exact test

Table S3. Differentially abundant microbes between responders and non-responders in the pre-BCG group (N=29)

Microbes	$\log_2$(Fold change)	$-\log_{10}$(adjusted *P*)	Enriched in
s__*Campylobacter ureolyticus*	21.37128325	5.010185526	Responders
s__*Bifidobacterium bifidum*	20.38109033	4.616845144	Responders

Table S4. Comparison of responders and non-responders for the post-BCG samples from patients with BCa (N=58)

	Responders (n=39)	Non-responders (n=19)	*p* value
Age (yrs), median (IQR)	73 (62, 82)	78 (68, 84)	0.1832[a]
Antibiotics usage (1 month)	1 (2.6%)	2 (10.5%)	0.2475[c]
Smoking	20 (51.3%)	8 (42.1%)	0.5827[c]
Gender, n (%)			>0.9999[c]
Male	31 (79.5%)	15 (78.9%)	
Female	8 (20.5%)	4 (21.1%)	
Grade, n (%)			0.3179[c]
Low	2 (5.1%)	3 (15.8%)	
High	37 (94.9%)	16 (84.2%)	
Recurrence, n (%)			**<0.0001**[c]
Recurred	0	17 (89.5%)	
Not recurred	39 (100%)	2 (10.5%)	
Progression, n (%)			**0.0314**[c]
Progressed	0	3 (15.8%)	
Not progressed	39 (100%)	16 (84.2%)	

* Statistics

[a] Unpaired parametric t-test

[b] Mann-Whitney U test

[c] Fisher's exact test

Table S5. Differentially abundant microbes at the genus level between responders and non-responders in the post-BCG group (N=58).

Microbes	$\log_2$(Fold change)	$-\log_{10}$(adjusted *P*)	Enriched in
g__*Prauserella*	11.02408465	4.512088178	Responders
g__*Rubrobacter*	7.385173028	3.269619668	Responders
g__*Marinococcus*	5.181322458	1.462785683	Responders
g__*Gluconacetobacter*	4.990624785	1.528931256	Responders
g__*Salmonella*	4.903388546	3.705370888	Responders
g__*Providencia*	4.814212602	2.258773969	Responders
g__*Sodalis*	4.734689504	2.589454454	Responders
g__*Trabulsiella*	4.720638591	3.7633223	Responders
g__*Klebsiella*	4.336545638	5.08778847	Responders
g__*Photorhabdus*	4.274696416	2.059008071	Responders
g__*Morganella*	3.979910534	1.637892802	Responders
g__*Citrobacter*	3.828595042	2.051359229	Responders
g__*Candidatus Blochmannia*	3.529238666	1.328078519	Responders
g__*Halochromatium*	3.265354752	1.539856638	Responders
**g__*Bifidobacterium***	3.079999457	1.539856638	Responders
g__*Planomicrobium*	2.759671276	1.328078519	Responders
g__*Erwinia*	2.36057155	1.972213803	Responders
g__*Enhydrobacter*	-2.021291464	1.930773026	Non-responders
g__*Veillonella*	-2.975684493	1.731111319	Non-responders
g__*Alicycliphilus*	-3.86964987	2.307995984	Non-responders
g__*Virgibacillus*	-5.65069365	6.178272422	Non-responders

Table S6. Differentially abundant microbes at the species Level between responders and non-responders in post-BCG group (N=58).

Microbes	$\log_2$(Fold change)	$-\log_{10}$(adjusted *P*)	Enriched in
s__*Prauserella rugosa*	11.02408465	4.512088178	Responders
s__*Klebsiella oxytoca*	5.431322691	1.755988355	Responders
s__*Salmonella enterica*	4.903215849	3.705370888	Responders
s__*Morganella morganii*	4.619418335	2.164005354	Responders
s__*Trabulsiella farmeri*	4.225860266	1.718626092	Responders
s__*Bacteroides coprophilus*	3.898261367	1.772719487	Responders
**s__*Bifidobacterium breve***	3.709381395	1.856693681	Responders
**s__*Bifidobacterium adolescentis***	3.208974442	1.718626092	Responders
**s__*Bifidobacterium longum***	2.993892886	1.488912582	Responders
s__*Streptococcus infantis*	-4.173576777	2.797769527	Non-responders
