# Supplementary material for: Differential Urinary Microbiome and Its Metabolic Footprint in Bladder Cancer Patients Following BCG Treatment
Source: Int J Mol Sci. 2024 Oct 17;25(20):11157. doi: 10.3390/ijms252011157 (PMC11508893; doi:10.3390/ijms252011157)
Supplement: Supplementary file 1 [file ijms-25-11157-s001.zip › #Supplementary Figures.pdf]

Supplementary Figures

Figure S1: Heatmap comparisons at the species level and urine metabolite analysis between benign and BCa groups, along with a schematic of the toluene degradation pathway.

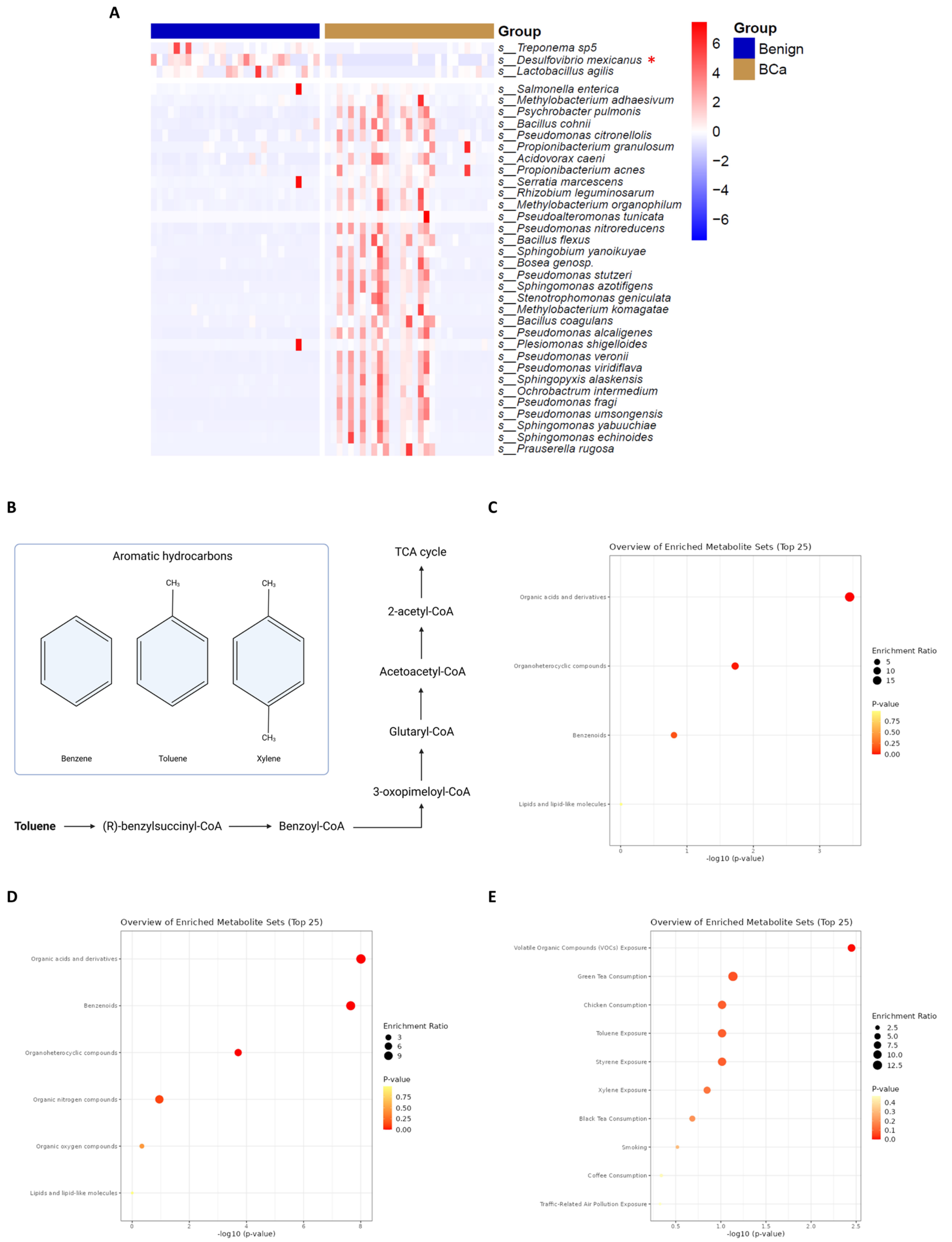

Figure S2. Comparative analysis among benign, pre-BCG, and post-BCG samples in male patients only

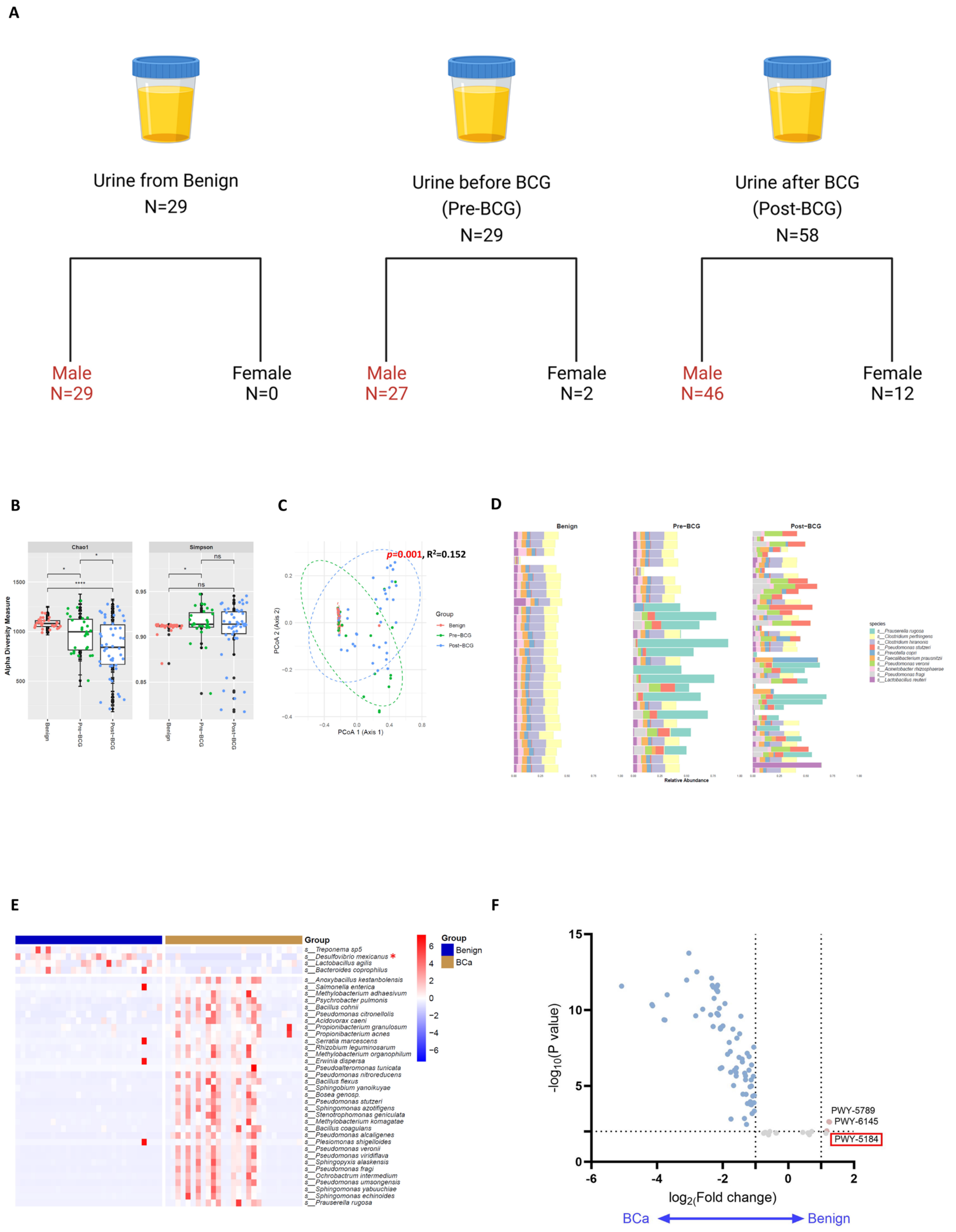

Figure S3. Comparative analysis between matched pre-BCG and post-BCG samples

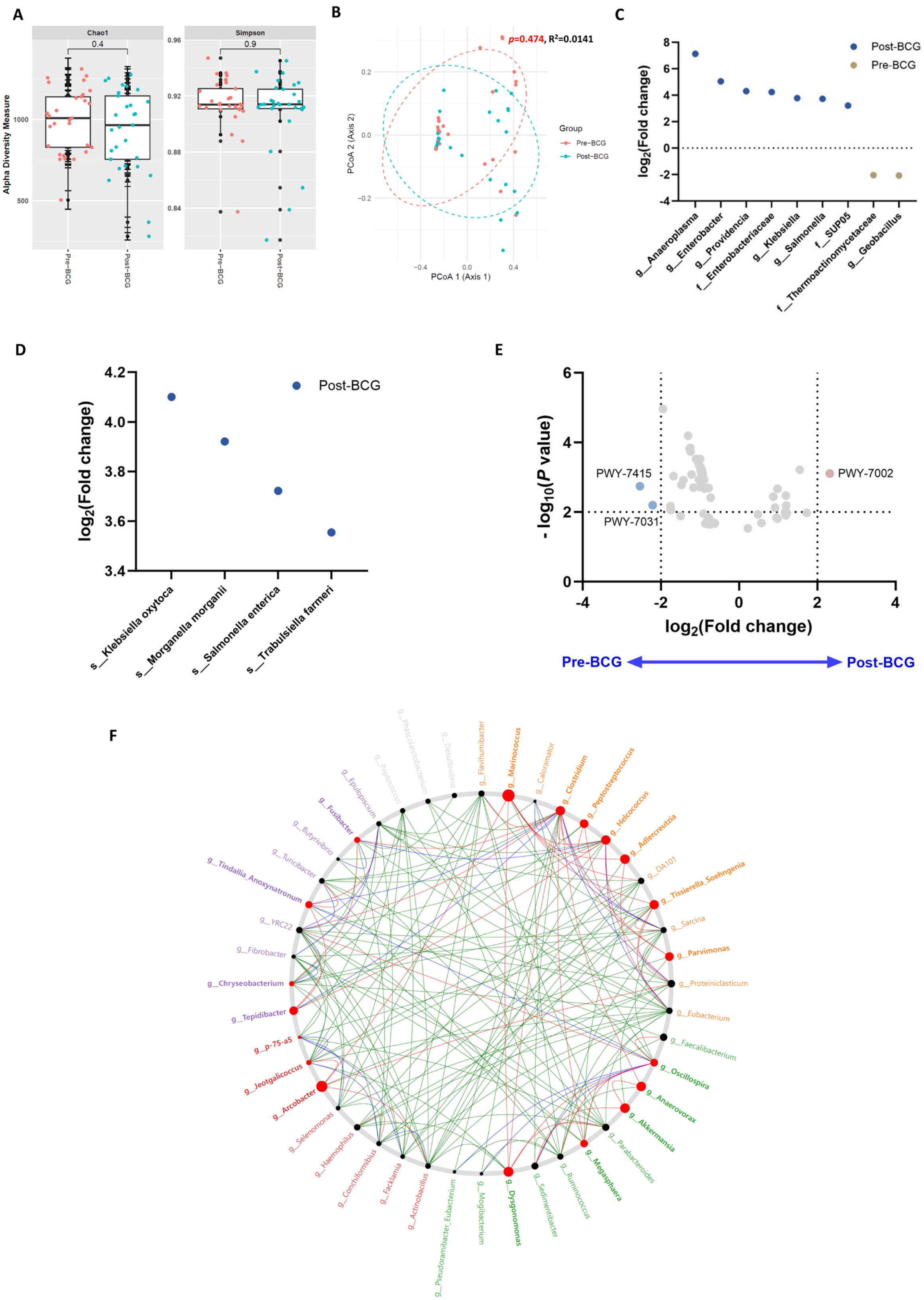

Figure S4. Comparative analysis based on response and gender in the pre-BCG and post-BCG groups

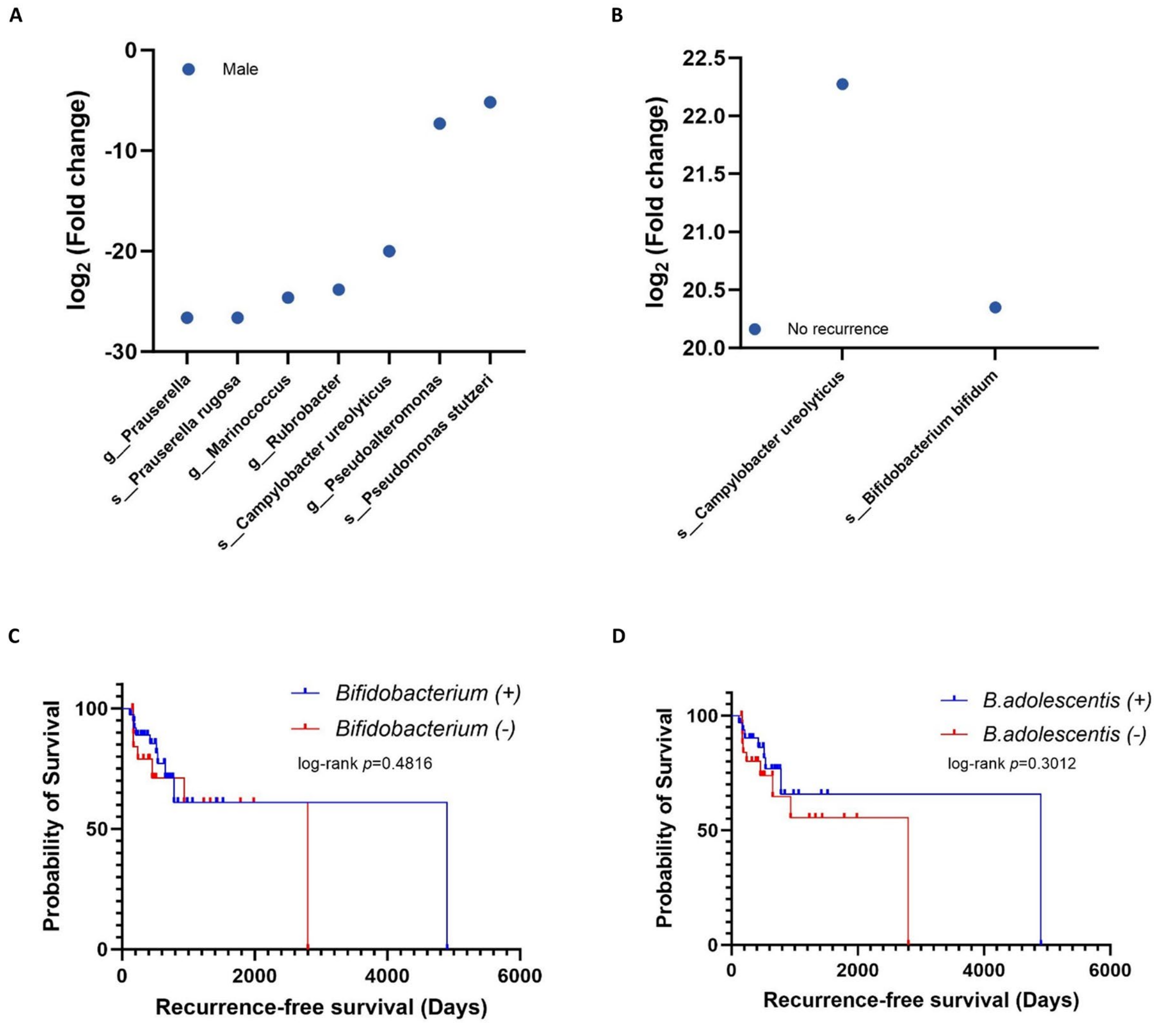

Figure S5. Comparative analyses based on tumor grade and recurrence in the post-BCG group

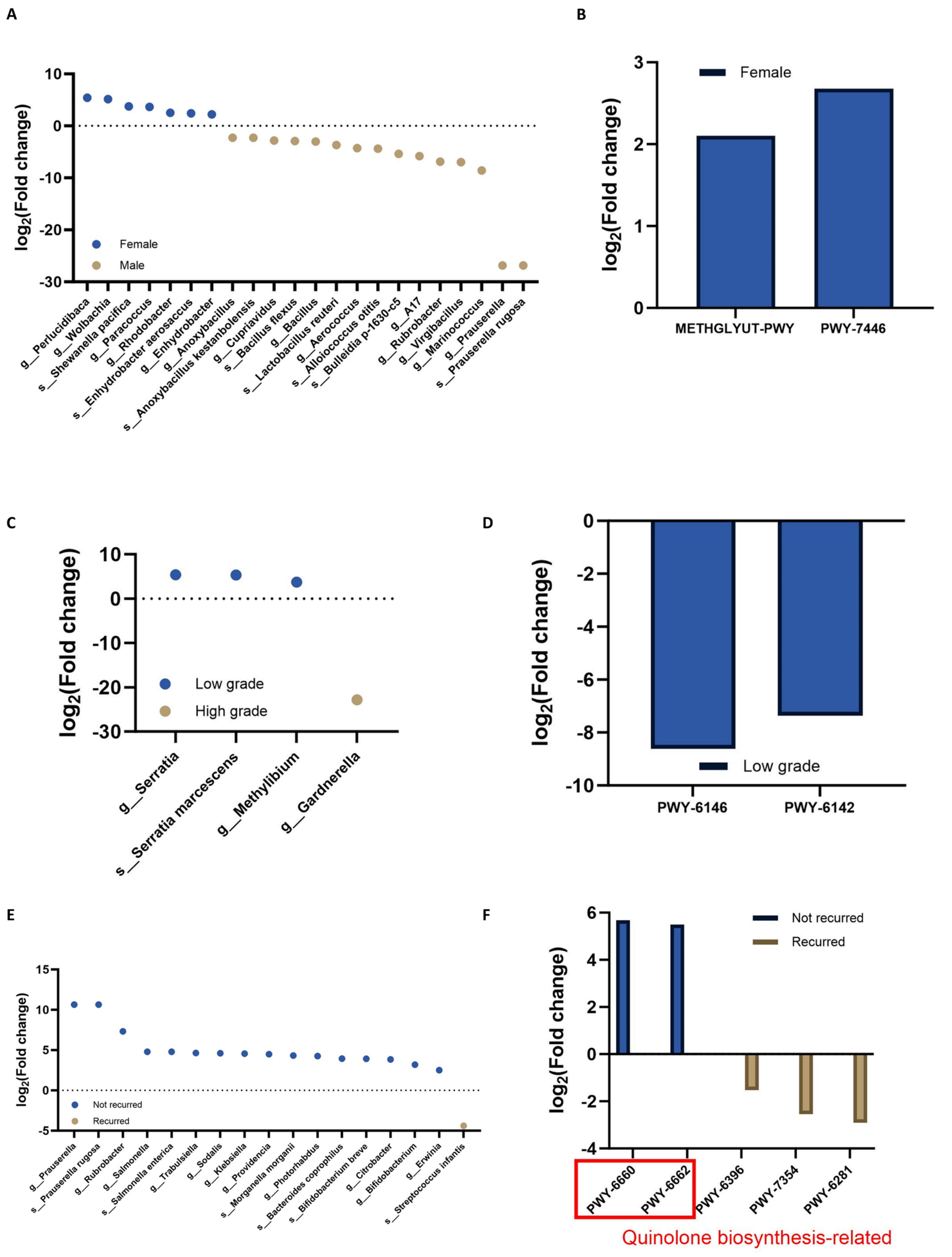

Figure S6. Heatmap illustrating variations in microbial composition correlated with progression status in the post-BCG cohort

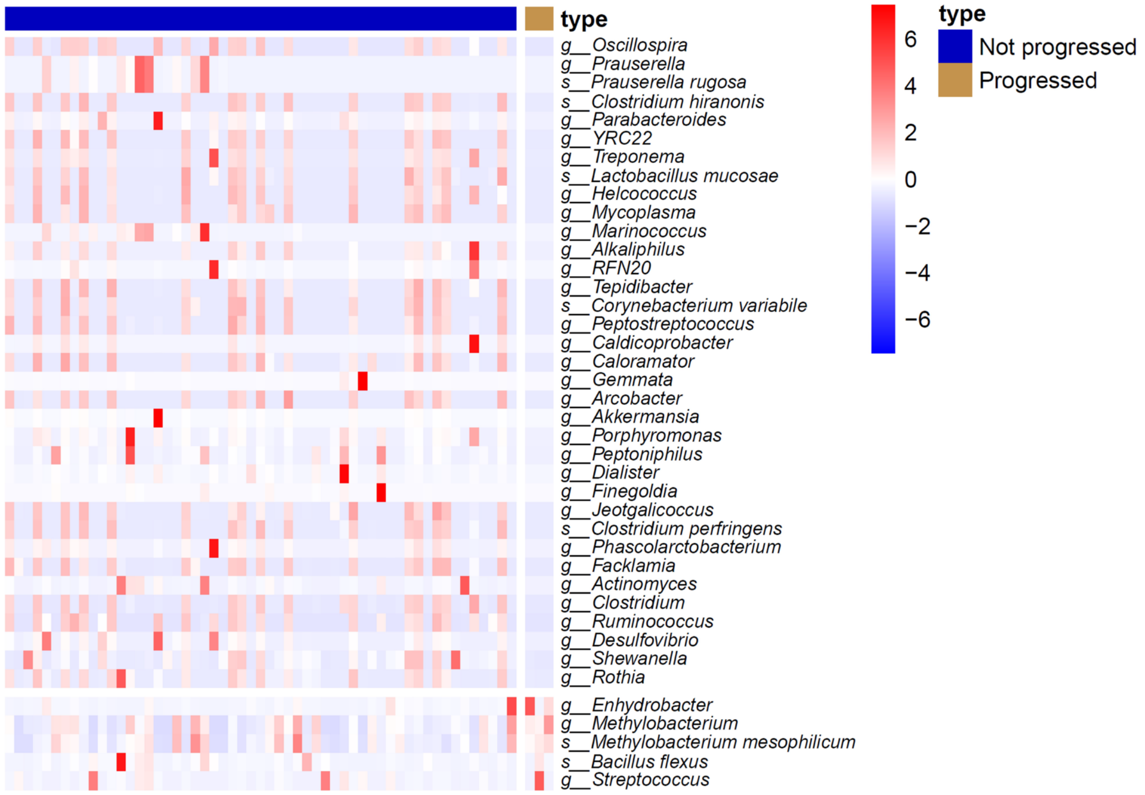

Figure S7: ROC curve for the prediction model of malignancy and response to BCG treatment

A

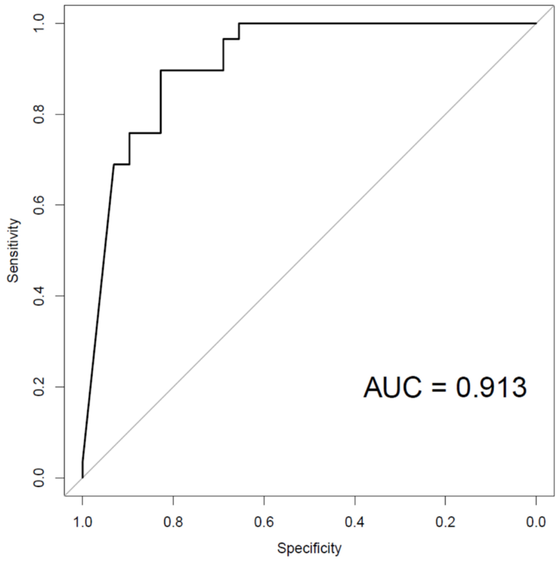

B

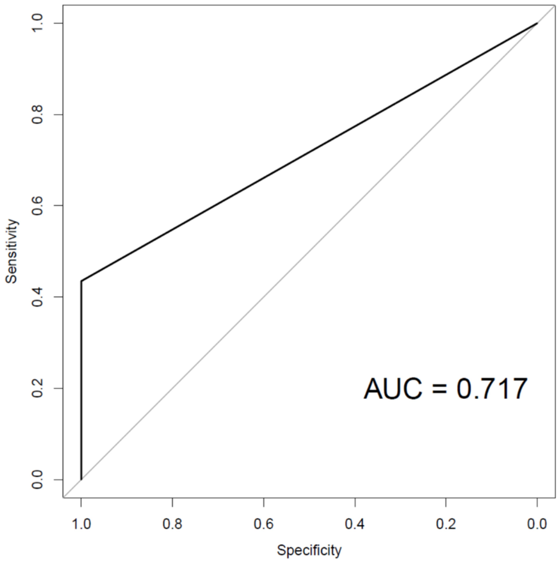

C

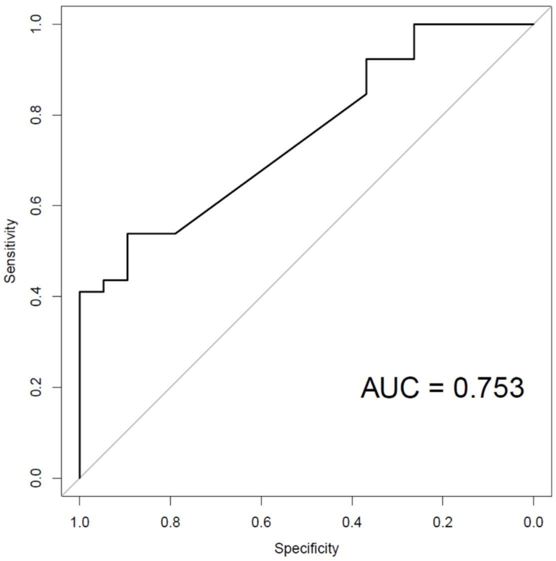

Supplementary Figure Legend

Figure S1: Heatmap comparisons at the species level and urine metabolite analysis between benign and BCa groups, along with a schematic of the toluene degradation pathway.

Figure S1A: Heatmap displaying microbial composition variations among groups. Colors represent different groups: blue for benign (n=29), and gold for BCa (n=29). Species selected by DESeq2 with *p* value<0.05 and $|\log_2(\text{Fold change})|>2$ for their differential abundance are annotated.

Figure S1B: Overview of aromatic hydrocarbons and the toluene degradation VI (anaerobic) pathway (PWY-5184). This pathway depicts the degradation of toluene into the TCA cycle.

Figure S1C: Enriched metabolite sets in BCa patients compared to benign patients, identified through a hypergeometric test in the ORA tool of MetaboAnalyst 6.0. These metabolite sets are categorized under the 'super-class' of chemical structures in the metabolite set library.

Figure S1D: Enriched metabolite sets in benign patients compared to BCa patients, identified through a hypergeometric test in the ORA tool of MetaboAnalyst 6.0. These metabolite sets are categorized under the 'super-class' of chemical structures in the metabolite set library.

Figure S1E: Enriched metabolite sets in benign patients compared to BCa patients, identified through a hypergeometric test in the ORA tool of MetaboAnalyst 6.0. These metabolite sets are included under 'other types' in the metabolite set library.

Figure S2. Comparative analysis among benign, pre-BCG, and post-BCG samples in male patients only

Figure S2A: Schematic illustration of the study design, utilizing urine samples from male patients (N=75).

Figure S2B: Comparison of Chao1 and Simpson indices of alpha diversity among benign (n=29), pre-BCG (n=27), and post-BCG (n=46) groups. Differences between groups were analyzed using the Wilcoxon test. Significance levels are indicated as follows: **p*<0.05, ***p*<0.01, ****p*<0.001, *****p*<0.0001.

Figure S2C: Beta diversity analysis among benign (n=29), pre-BCG (n=27), and post-BCG (n=46) groups using Bray–Curtis distance. The significance of beta diversity was determined by PERMANOVA with 999 permutations.

Figure S2D: Bar graph depicting the microbial composition of each group, highlighting and labeling the top 10 most abundant species.

Figure S2E: Heatmap displaying microbial composition variations among groups. Colors represent different groups: blue for benign (n=29), and gold for BCa (n=27). Genera selected by DESeq2 with *p* value<0.05 and $|\log_2(\text{Fold change})|>2$ for their differential abundance are

annotated.

Figure S2F: Volcano plot of differentially abundant metabolic pathways between benign (n=29) and BCa (n=27) groups. A positive fold change indicates enrichment in the benign group; a negative fold change indicates the opposite. Metabolic pathways selected by EdgeR with -$\log_{10}$(p value)>1 and $\log_2$(Fold change)≧2 for their differential abundance are annotated.

Figure S3. Comparative analysis between matched pre-BCG and post-BCG samples

Figure S3A: Comparison of Chao1 index of alpha diversity between pre-BCG and post-BCG samples in responders (n=23). Differences between groups were analyzed using the Wilcoxon test.

Figure S3B: Beta diversity analysis of pre-BCG and post-BCG samples in responders (n=23) using Bray–Curtis distance. The significance of beta diversity was determined by PERMANOVA with 999 permutations.

Figure S3C: Analysis of differential microbial abundance at family and genus levels in matched samples from the paired cohort (n=29). Microbes more abundant in the post-BCG group are highlighted in blue, while those in the pre-BCG group are gold. Microbes selected by DESeq2 with p value<0.05 and |$\log_2$(Fold change)|>2 for their differential abundance are annotated.

Figure S3D: Analysis of differential microbial abundance at the species level in matched samples from the paired cohort (n=29). Microbes more abundant in the post-BCG group are highlighted in blue, while those in the pre-BCG group are gold. Microbes selected by DESeq2 with p value<0.05 and |$\log_2$(Fold change)|>2 for their differential abundance are annotated.

Figure S3E: Volcano plot of differentially abundant metabolic pathways between paired pre-BCG (n=29) and post-BCG (n=29) groups. A positive fold change indicates enrichment in the post-BCG group; a negative fold change indicates the opposite. Metabolic pathways selected by EdgeR with -$\log_{10}$(p value)>2 and $\log_2$(Fold change)>2 for their differential abundance are annotated.

Figure S3F : Identification of potential 'driver microbes' affecting BCG treatment response in the paired cohort. This analysis compares pre-BCG (n=29) and post-BCG (n=29) groups. Node sizes correspond to scaled NESH scores, with nodes colored red indicating an increase in betweenness from pre-BCG to post-BCG microbiomes. Large red nodes signify key driver taxa in BCG treatment, with their names in bold. Line colors indicate connections: red for associations unique to post-BCG microbiomes, green for pre-BCG exclusive associations, and blue for those present in both groups.

Figure S4. Comparative analysis based on response and gender in the pre-BCG and post-BCG groups

Figure S4A: Differential microbial abundance at genus and species levels in the pre-BCG group

(n=29). Microbes enriched in males are in blue. Microbes selected by DESeq2 with *p* value<0.05 and $|\log_2(\text{Fold change})|>2$ for their differential abundance are annotated.

Figure S4B: Differential microbial abundance at species levels in the pre-BCG group (n=29). Microbes more abundant in patients without recurrence are in blue. Microbes selected by DESeq2 with *p* value<0.05 and $|\log_2(\text{Fold change})|>2$ for their differential abundance are annotated.

Figure S4C: Kaplan-Meier survival curve showing RFS based on the presence of the *Bifidobacterium* genus in the post-BCG group (n=58), analyzed using the log-rank test.

Figure S4D: Kaplan-Meier survival curve showing RFS based on the presence of *B. adolescentis* in the post-BCG group (n=58), analyzed using the log-rank test.

Figure S5. Comparative analyses based on tumor grade and recurrence in the post-BCG group

Figure S5A: Differential microbial abundance at genus and species levels in the post-BCG group (n=58). Microbes enriched in females are in blue, and those enriched in males are in gold. Microbes selected by DESeq2 with *p* value<0.05 and $|\log_2(\text{Fold change})|>2$ for their differential abundance are annotated.

Figure S5B: Comparison of metabolic pathway abundance between females and males in the post-BCG group (n=58). Pathways more abundant in females are in blue. Metabolic pathways selected by EdgeR with $-\log_{10}(p\text{ value})>2$ and $\log_2(\text{Fold change})>2$ for their differential abundance are annotated.

Figure S5C: Analysis of differential microbial abundance at genus and species levels in the post-BCG group (n=58). Microbes more abundant in patients with low-grade tumors are highlighted in blue, while those in high-grade patients are in gold. Microbes selected by DESeq2 with *p* value<0.05 and $|\log_2(\text{Fold change})|>2$ for their differential abundance are annotated.

Figure S5D: Comparison of metabolic pathway abundance according to tumor grade in the post-BCG group (n=58). Pathways more prevalent in low-grade patients are highlighted in blue. Metabolic pathways selected by EdgeR with $-\log_{10}(p\text{ value})>2$ and $\log_2(\text{Fold change})>2$ for their differential abundance are annotated.

Figure S5E: Differential microbial abundance at genus and species levels in the post-BCG group (n=58). Microbes more abundant in patients without recurrence are in blue, while those in patients with recurrence are in gold. Microbes selected by DESeq2 with *p* value<0.05 and $|\log_2(\text{Fold change})|>2$ for their differential abundance are annotated.

Figure S5F: Comparison of metabolic pathway abundance according to recurrence in the post-BCG group (n=58). Pathways more prevalent in patients without recurrence are highlighted in blue, while those more abundant in patients with recurrence are in gold. Metabolic pathways selected by EdgeR with $-\log_{10}(p\text{ value})>2$ and $\log_2(\text{Fold change})>2$ for their differential abundance are annotated.

Figure S6. Heatmap illustrating variations in microbial composition correlated with progression status in the post-BCG cohort

Colors represent different groups: blue for patients without progression (n=55), and gold for patients with progression (n=3). Genera selected by DESeq2 with *p* value<0.05 and $|\log_2(\text{Fold change})|>2$ for their differential abundance are annotated.

Figure S7: ROC curve for the prediction model of malignancy and response to BCG treatment

Figure S7A: The logistic regression-based model incorporated bacterial presence and abundance data, focusing on the genus *Thauera* and *Desulfovibrio mexicanus* from benign and pre-BCG urine samples, to make prediction for malignancy. Area under ROC curve was calculated.

Figure S7B: The logistic regression-based model incorporated bacterial presence and abundance data, focusing on the *C. ureolyticus and B. bifidum* from pre-BCG urine samples, to make prediction for the response to BCG treatment. Area under ROC curve was calculated.

Figure S7C: The logistic regression-based model incorporated bacterial presence and abundance data, focusing on the *Bifidobacterium* genus and specific species (*B. adolescentis, B. breve,* and *B. longum*) from post-BCG urine samples, to make prediction for the response to BCG treatment. Area under ROC curve was calculated.
